# An Alkaline Protease from *Bacillus pumilus* MP 27: Functional Analysis of Its Binding Model toward Its Applications As Detergent Additive

**DOI:** 10.3389/fmicb.2016.01195

**Published:** 2016-08-03

**Authors:** Mehak Baweja, Rameshwar Tiwari, Puneet K. Singh, Lata Nain, Pratyoosh Shukla

**Affiliations:** ^1^Enzyme Technology and Protein Bioinformatics Laboratory, Department of Microbiology, Maharshi Dayanand UniversityRohtak, India; ^2^Division of Microbiology, Indian Agricultural Research InstituteNew Delhi, India

**Keywords:** *Bacillus pumilus*, protease, surfactant, de-staining, molecular modeling, docking, detergent

## Abstract

A proteolytic strain of *Bacillus pumilus* MP 27 was isolated from water samples of Southern ocean produced alkaline protease. Since protease production need expensive ingredients, an economically viable process was developed by using low cost carbon source, wheat straw, supplemented with peptone. This protease was active within temperature ranges 10–70°C at pH 9. This process was optimized by response surface methodology using a Box Bekhman design by Design Expert 7.0 software that increased the protease activity to 776.5 U/ml. Moreover, the enzyme was extremely stable at a broad range of temperature and pH retaining 69% of its activity at 50°C and 70% at pH 11. The enzyme exhibited excellent compatibility with surfactants and commercial detergents, showing 87% stability with triton X-100 and 100% stability with Tide commercial detergent. The results of the wash performance analysis demonstrated considerably good de-staining at 50 and 4°C with low supplementation (109 U/ml). Molecular modeling of the protease revealed the presence of serine proteases, subtilase family and serine active site and further docking supported the association of catalytic site with the various substrates. Certainly, such protease can be considered as a good detergent additive in detergent industry with a possibility to remove the stains effectively even in a cold wash.

## Introduction

In recent years, significant advancements in agriculture, industrial, and biotechnological fields, has fueled the search of microorganisms with novel characteristics that can be utilized in scientific and industrial applications. Researchers constantly look for geographical locations with extreme conditions where microbes survive. Cold adapted regions like Antarctic (Chong et al., 2015) Southern ocean, Indian Ocean (Fulzele et al., 2011), etc. have proven to be excellent habitats in search of novel microbes. The metabolic product such as enzymes (green chemicals), produced by them offer advantages over the use of conventional chemical catalysts for various reasons, e.g., high catalytic activity and substrate specificity (Rai and Mukherjee, 2011). These microbes have potential to quench the constant need for biomolecules, active at extreme conditions. Enzymes like protease, lipases, amylases, etc. studied from different genera of cold adapted microbes are already used in the fields of agricultural, medical, molecular, and environmental biotechnology, but usage is still in the infancy.

The Antarctic experiences severe environmental conditions like extreme cold, broad temperature fluctuations, abrupt chemical gradients and low carbon and nitrogen supply. The Antarctic habitats are regarded as ecosystems of low productivity because of their oligotrophic situation and the low temperatures. In winter, the level of photosynthetic active radiation is low and energy supplies are dramatically decreased. However, during the summer season, a period of high irradiance and mild temperatures, maximal biological C- and N-fixation rates are achieved, increasing carbon and nitrogen supply to the different Antarctic environments. Although the environment is inhospitable, there is a highly diverse microbial population in Antarctic soils and water samples (Niederberger et al., 2008). Thus, these environments constitute a suitable place for the isolation of bacteria that produce extremoenzymes.

Microbial proteases are multifunctional enzymes, which can catalyze hydrolysis of proteins to polypeptides, oligopeptides and finally to amino acids. Among different types (acidic, neutral, and alkaline) of proteases, alkaline proteases are the most commonly used industrial enzyme due to their relatively high activity and stability at high pH (Guleria et al., 2016). But often, production is low and optimization is needed to maximize the enzyme production. Optimization of major nutritional factors, i.e., sources of carbon, nitrogen, growth factors, and metal ions is instrumental in increasing the enzyme production and making the industrial process cost-effective and economically viable. The ‘one-variable-at-a-time’ approach allows optimization of nutritional/cultural factors, by changing one factor at a time, while keeping other variables constant. Conversely, the statistical response surface methodology (RSM) model is useful for studying the effect of several factors simultaneously influencing the enzyme production. Globally, proteases alone contribute 60% market share among all industrial enzymes, *Bacillus* being the major producer (Bouacem et al., 2015). Proteases are one of key constituent to improve wash performance in detergent formulation (Grbavcic et al., 2011). Most often, the detergent protease belongs to serine protease that acts by cleaving peptide bonds in proteins, in which serine serves as the nucleophilic amino acid at the active site of the enzyme. They catalyze the reaction using catalytic triad consisting of serine, histidine, and aspartate. Each amino acid in the catalytic triad has the specific role during catalysis. These three amino acids may not be closely situated in the sequence but they form the triad after protein folding.

The current study is intended to scrutinize the proteolytic bacteria isolated from Southern ocean, a niche for extremophiles. The main focus of our study is to optimize the cultural and nutritional conditions to produce the maximum level of protease and to unravel the functional characteristics of protease enzyme to find its suitability as detergent additive.

## Materials and Methods

### Sample Collection and Isolation of Microorganisms

The water samples were collected from seven different stations of Southern ocean- 37°S 57°E, 39°S 57°30 E, 41°S 57′ 30°E, 45°S 57°30, 48°S 57°30 E, 52°S 57°30 E, 60°S 57°30 E, 60°S 52°59 E, 50°S 47°E, 48°S 47°E, 46°S 47°E, 46°S 47°E, 44°S 47°E, 42°S 47°E, 40°E 47°S. The samples were kept at 4°C at the time of transportation and at -20°C in lab.

The water samples were centrifuged at 10,000 rpm for 15 min and the pellet was vortexed and inoculated on medium containing peptone 0.2%, yeast extract 0.2%, glucose 0.1%, KH_2_PO_4_ 0.002%, MgSO_4_.7H_2_O 0.005% and casein hydrolysate 0.2% and incubated at 20°C in an incubator shaker for 15 days. Finally, the inoculum was spread plated on the same medium and different morphotypes isolated were purified further.

### Screening of Bacterial Isolates for Protease Activity

The isolates were screened qualitatively for protease production on minimal media (Peptone 0.5%, beef extract 0.3%, sodium chloride 0.5%, agar 2%) containing gelatin, casein, and skim milk (1%) as a protein source. Plates were incubated at 20°C for 24 h. After incubation, plates were flooded with 5 ml of mercuric chloride reagent. The hydrolytic zones were observed around the bacterial colonies. On the basis of the qualitative assay, the most promising isolates were selected for further studies.

The positive isolates were further screened quantitatively to assess their protease production potential. Submerged fermentation was carried out for protease production in a medium containing- glucose 10 g/l, peptone 5 g/l, yeast extract 5 g/l, KH_2_PO_4_ 1 g/l, and MgSO_4_.7H_2_O 0.2 g/l at pH 8.0 adjusted with 20% w/v sodium carbonate (Kuddus and Ramteke, 2011). The overnight grown bacterial culture [1% (v/v)] was inoculated in the protease production medium. The flasks were incubated at 30°C in incubator shaker at 150 rpm. The crude extracellular enzyme was collected after centrifugation at 10,000 *g* for 10 min and was assayed for protease activity at different pH and temperature.

### Protease Assay

Protease activity was determined by incubating 250 μl of azocasein [1% (w/v)] with 150 μl of crude enzyme for 20 min at 37°C as described by Sarath et al. (1989) and Pranaw et al. (2013). The assay temperature was optimized by incubating the enzyme at temperature ranging from 10–70 degrees.

### Identification of Strain

The genomic DNA was extracted from bacterial culture using Zymo Research Fungal/Bacterial DNA MicroPrep^TM^ following the manufacturer’s standard protocol. The 16S rRNA gene was amplified by PCR using a set of universal primers pA (5′-AGA GTT TGA TCC TGG CTC AG-3′) and pH (5′-AAG GAG GTG ATC CAG CCG CA-3′; Edwards et al., 1989). The PCR products were purified and sequenced by Xcelris Labs Limited, Ahmedabad, India. The sequence was submitted and accession number was provided by GenBank.

### Optimization of Protease Production Medium

#### Selection of Significant Parameters in Protease Production

The selection of significant parameters influencing protease production potential of the isolate was initially screened by one-variable-at-a-time approach. Each parameter was sequentially optimized stating with carbon source followed by nitrogen source, co-factors and surfactants. Six carbon source (1%), including paddy straw, wheat straw, wheat bran, baggase, glucose and sucrose, five nitrogen source (0.1%) peptone, yeast extract, ammonium chloride, soybean meal and urea, five metal ions (0.1%) including CaCl_2_, MnSO_4_, CuSO_4_, FeSO_4_ and ZnSO_4_ and four surfactants (0.1%) including Tween-20, Tween-80, Triton X-100 and SDS were added at their respective concentrations in minimal medium (glucose 10 g/l, KH_2_PO_4_ 1 g/l, and MgSO_4_.7H_2_O 0.2 g/l) and protease activity was evaluated after incubation at 30°C for 48 h in incubator shaker. The agro residual substrates used during the carbon source optimization were first washed with distilled water, air dried, chopped (particle size 0.5–1 cm) and stored at room temperature until further use.

#### Experimental Design and Protease Production

After selection of significant parameters, optimization of protease production was done by Box Bekhman design using Design Expert 7.0 (Stat- Ease, Inc., Minneapolis, MN, USA). The significant variables were A wheat straw (C source), B peptone (N source), CaCl_2_ (Co factor) and D Tween-20 (surfactant), each was assessed in a range as is shown in **Table 1.** A total of 27 experiments were performed in triplicates. The experiment design along with experimental and predicted values is depicted in **Table 2.**

**Table 1:** Experimental range and levels of the independent variables to optimize media components for protease production by *Bacillus pumilus* MP 27.

Factors	Independent variables	Range and levels		
		
		-1	0	1
A	Carbon source	1	3	5
B	Nitrogen source	0.1	0.3	0.5
C	Co factor	0.1	0.3	0.5
D	Surfactant	0.1	0.3	0.5


**Table 2:** Results of Box Bekhman using four independent variables and three center points showing predicted and observed response of protease production by *B. pumilus* MP 27 under submerged fermentation.

Trials	A (%)	B (%)	C (%)	D (%)	Predicted value (U/ml)	Observed value (U/ml)
1	1	0.1	0.3	0.3	184.308	130.5
2	5	0.1	0.3	0.3	619.563	680.87
3	1	0.5	0.3	0.3	422.781	396.99
4	5	0.5	0.3	0.3	583.536	672.86
5	3	0.3	0.1	0.1	494.09	549.48
6	3	0.3	0.5	0.1	369.92	399.25
7	3	0.3	0.1	0.5	542.215	548.4
8	3	0.3	0.5	0.5	507.875	488
9	1	0.3	0.3	0.1	277.107	232
10	5	0.3	0.3	0.1	747.987	655.91
11	1	0.3	0.3	0.5	542.932	590.5
12	5	0.3	0.3	0.5	668.152	668.84
13	3	0.1	0.1	0.3	395.51	380
14	3	0.5	0.5	0.3	427.844	380.41
15	3	0.1	0.1	0.3	247.365	250.38
16	3	0.5	0.5	0.3	417.479	388.57
17	1	0.3	0.3	0.3	485.198	520
18	5	0.3	0.3	0.3	613.813	580.38
19	1	0.3	0.3	0.3	236.553	278.89
20	5	0.3	0.3	0.3	703.948	678.05
21	3	0.1	0.1	0.1	316.527	340.76
22	3	0.5	0.5	0.1	432.82	460.96
23	3	0.1	0.1	0.5	424.637	405.4
24	3	0.5	0.5	0.5	510.79	495.46
25	3	0.3	0.3	0.3	479.236	498.92
26	3	0.3	0.3	0.3	479.263	475.35
27	3	0.3	0.3	0.3	479.263	463.52


The experimental values were examined for significance of the model and model terms via Fisher’s test. The eminence of fit of the second-order polynomial model or the aptness of the model was conveyed by the coefficient of determination (*R*^2^) and the adjusted *R*^2^. The response surface plots were drafted from fitted polynomial equation and regression analysis was done in order to understand the relationship response and experimental level of each variable. The enzyme produced after the media optimization was further characterized.

### Evaluation of Alkaline Protease Stability with Different Physico-Chemical Factors

The effects of various physical and chemical factors were evaluated in order to assess the stability of protease. The stability was calculated after pre-incubating the enzyme up to 8 h at different temperatures (10–50°C). The effect of pH was evaluated from pH 7–11, using sodium phosphate buffer (pH 7.0–8.0) and glycine NaOH (pH 9.0–11.0) buffers (Tiwari et al., 2014) Furthermore, the stability of protease in the presence of surfactant like Tween-20, Tween-80, SDS, and Triton X-100 at 0.1% concentration for different time intervals was also checked (Gupta et al., 2005). The activity in the absence of surfactant was taken 100% and marked as control. The stability of the enzyme in the commercial detergent was also evaluated in 7 mg/ml concentration for 1 h and assayed for residual alkaline protease activity. The control was taken as 100% at Y-axis, i.e., in the absence of commercial detergent.

### Amplification of Alkaline Protease Gene

The genomic DNA was extracted from bacterial culture using Zymo Research Fungal/Bacterial DNA MicroPrep^TM^ following the manufacturer’s standard protocol. Isolated DNA was used as a template for amplification of alkaline protease gene. Based on the conserved nucleotide sequences, a pair of primers (Pro1 F: ATG TGC GTG AAA AAG AAA AAT GTG and Pro 1 R: TTA GTT AGA AGC TGC TTG AAC GTT was designed for amplifying alkaline protease encoding gene using the Primer Blast tool.

Amplification of protease gene was performed by 25 μl PCR reaction containing 2.5 μl 10x PCR buffer, 0.5 μl 10 mM dNTP, 0.5 μl 10 μM Forward primer, 0.5 μl μM reverse primer, 0.5 μl (3 U) Taq polymerse, 3 μl DNA template and 17.5 μl of nuclease free water. The amplification was done in a thermocycler with the following steps: 5 min primary denaturation at 95°C and followed by PCR cycles which comprises of denaturation at 95°C for 30 s, annealing at 53°C for 30 s, extension at 72°C for 1 min and final extension at 72°C for 10 min and held at 4°C. The amplified PCR products were electrophoresed in an agarose gel and visualized in UV radiation after staining with ethidium bromide (1 mg/ml). The product was purified using the Qiagen purification kit as per manufacturer’s instructions and sequenced.

### Molecular Docking

Modeling of the gene was performed by SWISS-MODEL an automated web server at http://swissmodel.expasy.org/ (Arnold et al., 2006; Guex et al., 2009; Kiefer et al., 2009; Biasini et al., 2014). Modeling of a protein refers to constructing an atomic-resolution model of the target protein from its amino acid sequence and an experimental three-dimensional structure of a related homologous protein template. The presence of functional domains in the gene was predicted by PRODOM server^1^ (Servant et al., 2002).

Docking of the modeled structure was performed with different substrate to analyze the substrate specificity and active site analysis. Ligands for the docking was retrieved from different sources, i.e., PDB and Pubchem. Hex 8.0.0 was taken to perform docking studies (Macindoe et al., 2010). It is an interactive Molecular Graphics Program for calculating and displaying feasible docking modes of pairs of protein and ligand molecules. It assumes ligands as rigid and binds it into the active sites or surface with optimizing the total energy. It uses Spherical Polar Fourier (SPF) correlations to accelerate the calculations and its one of the few docking programs which has built in graphics to view the result.

### Wash Performance Analysis

Cotton clothes (4 cm × 6 cm) were stained with tomato sauce (Heinz) and dried in oven. The detergent’s endoproteases were denatured by boiling the detergent solution at 100°C for 1 h. The stained cloth was treated with (1) Tide detergent (7 mg/ml) diluted in tap water, (2) enzyme (109 U) in water (3) detergent (7 mg/ml) supplemented with 109 U enzyme in tap water. The different mixtures were diluted in 50 ml of tap water and washed under shaking condition (200 rpm) for 15 min at 50, 20, and 4°C for 30 min.

## Results

### Isolation, Screening, and Identification of Bacteria

A total 28 bacterial isolates (MP 1 to MP 28) were obtained after enrichment, of them eight were showing protease activity by qualitative screening. The isolate MP 27 produced remarkable zone of hydrolysis on plates (**Table 3**) and was selected for further studies.

**Table 3:** Qualitative screening of proteolytic isolate MP 27 on different proteinaceous substrates.

Substrate	Colony diameter (d) (mm)	Diameter of zone of hydrolysis (D) (mm)	(D/d) (mm)
Casein	23	53	2.3
Gelatin	31	72	2.32
Skim milk	30	50	1.66


The isolate was identified as *Bacillus pumilus* based on 16S rRNA gene sequencing. The sequence was matched with the available sequence in the NCBI through BLAST, which showed a high homology (98%) with *B. pumilus* strain KL-052. The sequence was submitted and accession number was provided by GenBank *Bacillus* KX156953.

### Analysis of Protease Activity at Different Temperature and pH

**FIGURE 1:**
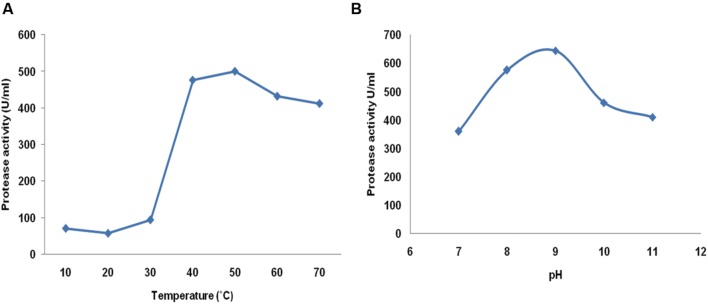
**Evaluation of enzyme activity at different **(A)** Temperature (10–70°C) and **(B)** pH (7–11) of *Bacillus pumilus* MP 27**.

Physical parameters play an important role for activity of any enzyme. All positive isolates were further screened for protease enzyme by quantitative means under submerged condition at 150 rpm at 30°C. Among eight isolates, isolate MP 27 showed maximum protease activity (456 U/ml) after 48 h of incubation. Also, growth was observed from 10 to 50°C, further increase in temperature inhibits the growth of the organism. The enzyme was evaluated for its protease activity at different temperatures during the protease assay. The enzyme was active over a broad range of temperature, however, the maximum activity was observed at 50°C. The enzyme was also active at 70°C so it reveals psychrothermotolerant nature of the enzyme (**Figure 1A**; Singh et al., 2011). The protease activity was also checked at different pH to deduce the nature of the protease enzyme. The highest protease activity was observed at 9.0 pH, further, the enzyme was active up to pH 11.0, retaining 63% of the activity which illustrated the alkali-tolerant nature of protease enzyme (**Figure 1B**). The psychrothermotolerant nature of this enzyme together with its alkaliphilic nature favors its application in detergent industry.

### Evaluation of Most Significant Parameters for Protease Production

**FIGURE 2:**
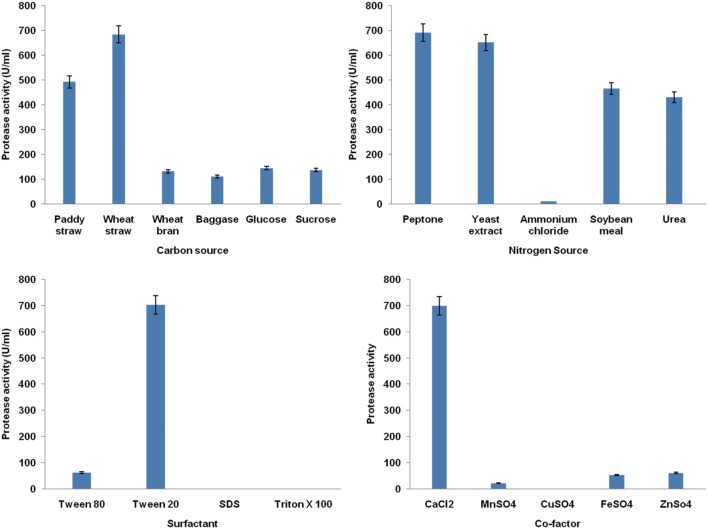
**Effect of media components (carbon source, nitrogen source, co-factors and surfactants) on protease production by *B. pumilus* 27 using one parameter at time approach**.

Generally, there is no defined medium specifically for protease production by different microbes. Each organism has their own specific requirements for maximization of protease production. In the current study, significant variables influencing protease production were selected by one parameter at a time approach. Among various carbon sources tested, the results indicated that different carbon sources have a variable impact on the production of extracellular protease. All tested carbon sources supported the growth of strain MP 27. However, wheat straw was found to be the best carbon source for maximum protease production as shown in **Figure 2.** Further, among nitrogen sources, peptone exhibited a prominent effect on the production ability resulting in higher protease activity (**Figure 2**). Also, both of the selected substrates are less costly which will reduce the cost of the production process.

Since cofactors are necessary for proper functioning of enzyme, several cofactors and surfactants were also evaluated. The CuSO_4_ inhibited the growth, whereas in the presence of calcium chloride, there were luxuriant growth and protease activity was shot up (**Figure 2**). Similarly, the presence of Tween-20 enhanced the protease activity (**Figure 2**).

### Statistical Optimization

The RSM, a statistical approach, was adopted to investigate the optimum levels of selected variables and interactions during protease production by *B. pumilus*. The variables contributing significantly to yield with maximum response were identified as, wheat straw (C source), peptone (N source), calcium chloride (Co-factor), and Tween-20 (surfactant). The quadratic model was constructed by Design expert using twenty seven trials of experiments. The design matrix and the corresponding results of Box Bekhman experiments were shown in **Table 2**, along with the predicted values. The experimental data fitted to a second-order polynomial equation and regression equation coefficients were calculated. The response, i.e., protease activity (Y) by *B. pumilus* MP27, can be expressed in terms of the following regression equation

Y(Response)=+479.26+149.00*A+50.61*B+39.63*C+46.52*D−68.63*A*B+84.69*A*C+86.39*A*D+34.44*B*C−7.53*B*D+22.46*C*D+55.56*A2−82.27*B2−24.94*C2+24.20*D2

Where, A, B, C, and D are carbon source, nitrogen source, cofactor, and surfactant, respectively.

**FIGURE 3:**
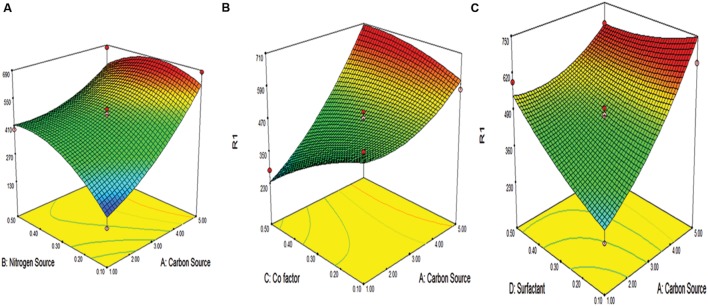
**Response surface plots of alkaline protease production showing interaction between **(A)** carbon and nitrogen source **(B)** carbon source and co factor **(C)** carbon source and surfactant**.

The results of the second order response surface model were analyzed by analysis of variance (ANOVA) as given in **Table 4.** The high *F*-value, i.e., 10.20 and non-significant lack of fit (13.10) for the model illustrates the significance of the model for protease production. The inference deduced from *p*-value shows that linear effects of the carbon source, nitrogen source, cofactor, surfactant, interactive effects of AB, AC, and AD and square effects of nitrogen source were significant for protease production. The interaction between the variables is shown in **Figure 3** by 3D models. The validation of the statistical model and regression equation were conducted by taking 4.98% wheat straw, 0.34% of peptone, 0.16% CaCl_2_ and 0.11% of Tween-20. When the cells were supplemented under these optimized conditions 67% increase in protease activity was observed (776.5 U/ml). This formulation is cost effective for protease production process, as compared with expensive carbon sources used by other researchers during protease production.

**Table 4:** Analysis of statistical optimization via ANOVA for the experiment.

Standard deviation	59.99
Mean	467.06
*R*^2^	0.9225
Adjusted *R*^2^	0.832
Predicted *R*^2^	0.5576
Coefficient of variance	12.84
*F*- value	10.2


The coefficient of determination (*R*^2^) for protease production *R*^2^ was 0.9225 (above 0.75 values designates fitness of the model). The value illustrates that the model is able to justify 92.25% of the variation in response. The ‘adequate precision value’ of 29.50 (>4 indicates a good fit of the model) suggests that the model can be used to navigate the design space. The interaction among the variables has been shown with the help of 3-D plots as shown in **Figure 3.**

It was observed from **Figure 3** that carbon source played a significant role in the protease activity. The carbon source and response was directly proportional to each other. As the carbon source increase, there was increase in response. Also, as the carbon source and other factors like nitrogen source, co-factor and surfactant increase, there is concomitant increase in response, i.e., protease activity.

### Evaluation of Stability of Alkaline Protease in Presence of Physico-Chemical Factors

**FIGURE 4:**
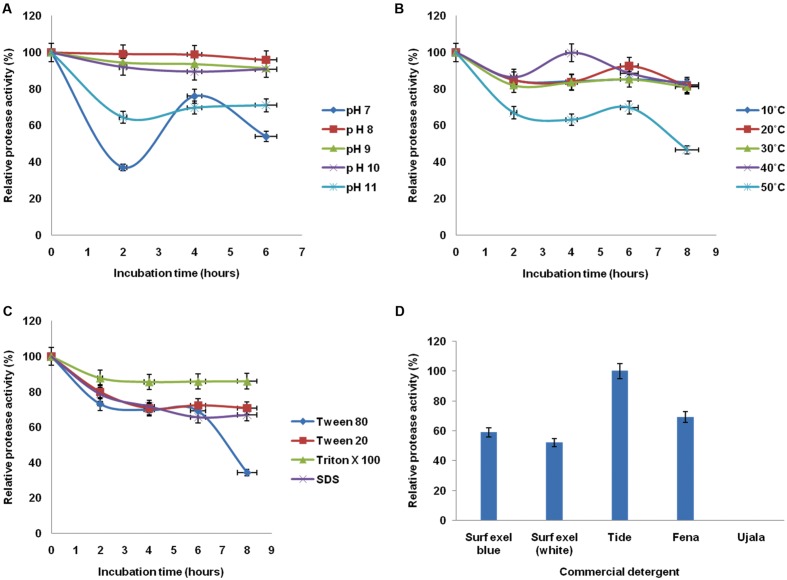
**Evaluation of stability of alkaline protease in presence of physico-chemical factors from *B. pumilus* MP 27 at different intervals **(A)** pH, **(B)** Temperature, **(C)** Surfactant, **(D)** Commercial detergent**.

The relative alkaline protease activity was evaluated for stability in a wide range of temperature, pH, surfactants, and commercial detergents at different time intervals. Since all these parameters essentially contribute in the application of de-staining, the result will help to determine its role in laundry industry. The enzyme exhibited an immense potential in retaining the stabilities at broad range. The stability of enzyme was seen 85.14% at 10°C whereas the maximum stability was observed at 40°C. The stability at 10 and 20°C is almost equivalent and thus the profile of 10°C is overlapped by 20°C. The stability was retained to significant level after 8 h of incubation (**Figure 4B**). The enzyme was quite stable in wide pH and temperature range deducing the psychro-thermoalkaline nature of protease.

Stability of extracellular protease at high pH was studied for its application in detergent industry. Highest stability of protease was observed at pH 8 (98.63%), but the enzyme was active up to pH 11.0, retaining 70.89% of the activity. Since the enzyme was active over a broad range of pH, i.e., from 7.0 to 11.0 which justifies the alkali-tolerant character of protease enzyme. The alkaline protease enzyme retained significant activity at pH 8 after 6 h of incubation (**Figure 4A**).

In addition to considerable temperature and pH stability the enzyme must be compatible to different surfactants and commercial detergents to work as a laundry additive. The potential of protease enzyme was assessed for its stability with different surfactants by pre incubating the enzyme with respective surfactant and commercial detergent. The protease showed excellent stability with different surfactants and commercial detergents. The enzyme supplemented with Triton X-100 found to retain 87.73% proteolytic activity (**Figure 4C**). Moreover, there was 100% relative protease activity after treatment with Tide as commercial detergent (**Figure 4D**). Indeed, the enzyme could be used as a detergent additive since it showed the good stabilities at different parameters essential for cost effective de-staining.

### Identification of Protease Gene

**FIGURE 5:**
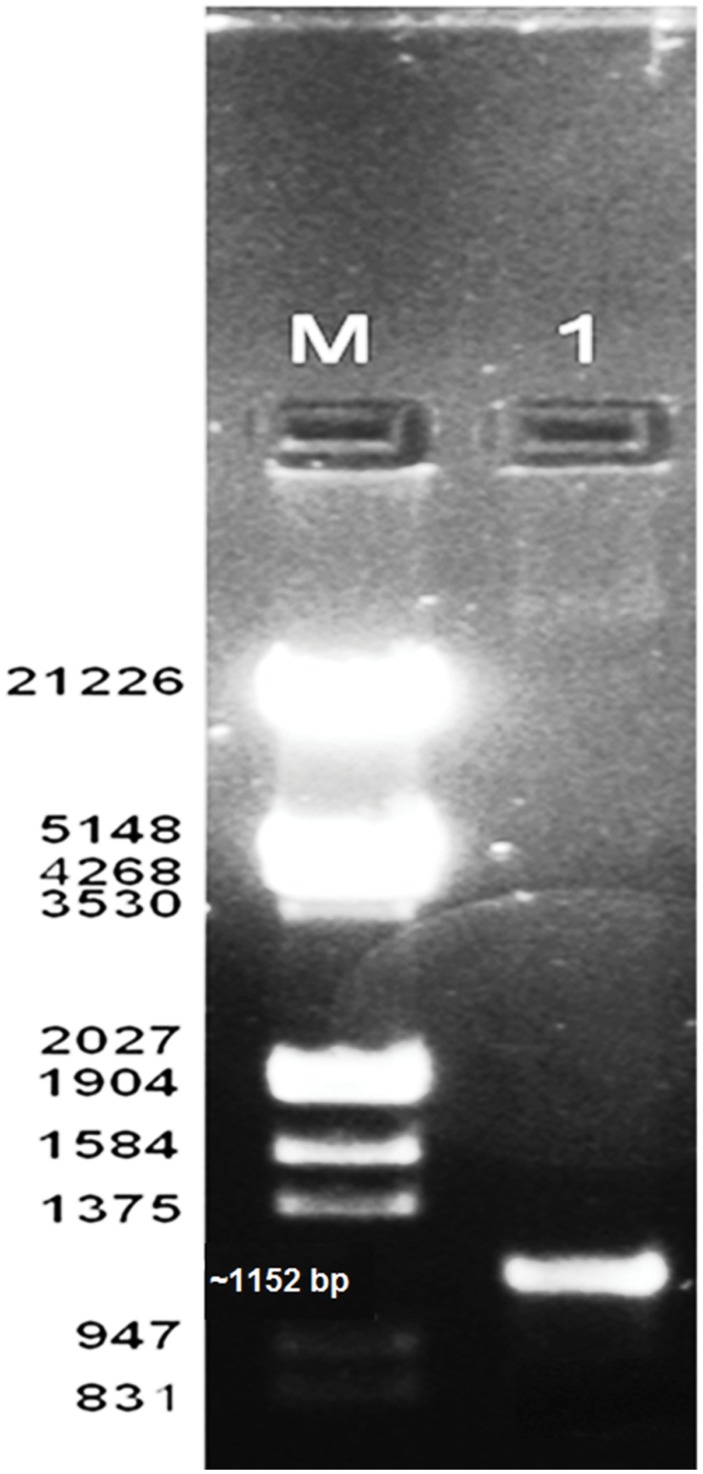
**Amplification of protease gene (M) Marker (1) amplified gene**.

After PCR amplification 1152 bp gene was amplified using the primers and submitted to GenBank (Accession no. KX431582) as shown in **Figure 5.** The sequence was matched with the available sequence in the NCBI through BLAST, which showed a high homology (97% homology with alkaline serine protease from *B. pumilus* TMS55). The gene sequence was translated and further modeled and docked with various substrates.

### Molecular Docking

The protein was modeled with swiss model and the PDB ID of similar alkaline proteinase from the mesophilic bacterium *Bacillus mesentericus* (1mee.1.A) was taken as template with 74.56% with query sequence and resolution of 2 Å. The model was further assessed by saves server^2^ Ramachandran plot of modeled protein contained 93.49% residue in the most favorable region, making it a stable model. Further analysis through ERRAT with overall quality factor of 91.643 supported the result.

**FIGURE 6:**
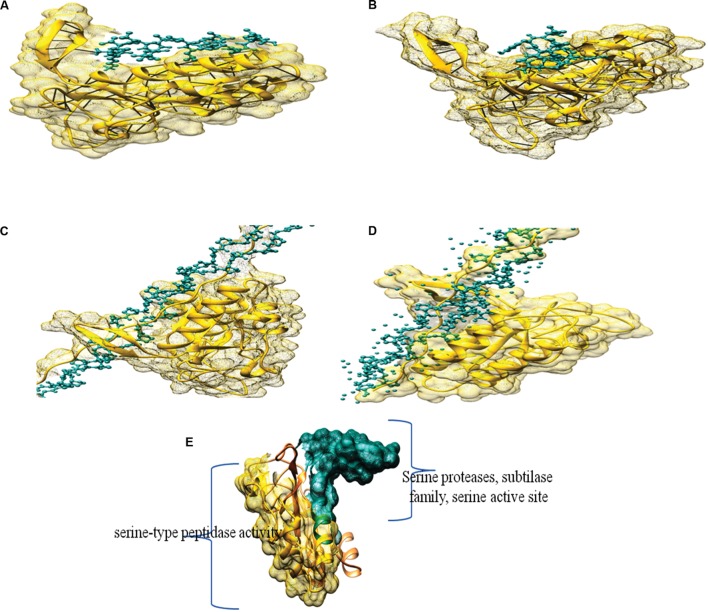
**Docking complex of modeled structure with **(A)** Casein, **(B)** Keratin, **(C)** Gelatin, **(D)** Azocoll, and **(E)** Domains of modeled structure from PRODOM**.

Molecular docking was performed by Hex 8.0.0 with modeled structure and ligand as Keratin, Azocoll, Casein and Gelatin as substrate (**Table 5**). Amongst all docking score gelatine showed superior binding energy of -609.97 (E-total) followed by Collagen -602.69 (E-total). The noteworthy thing about the docking results was all the substrate was docked in the Serine proteases, subtilase family, serine active site (**Figure 6**).

**Table 5:** Docking scores of modeled structure of protease docked with different substrate.

S.no	Ligand used docking with modeled structure of protease	Hex score (E-total)
1	Keratin	-341.53
2	Azocoll	-602.69
3	Casein	-427.67
4	Gelatin	-609.97


### Wash Performance Analysis

**FIGURE 7:**
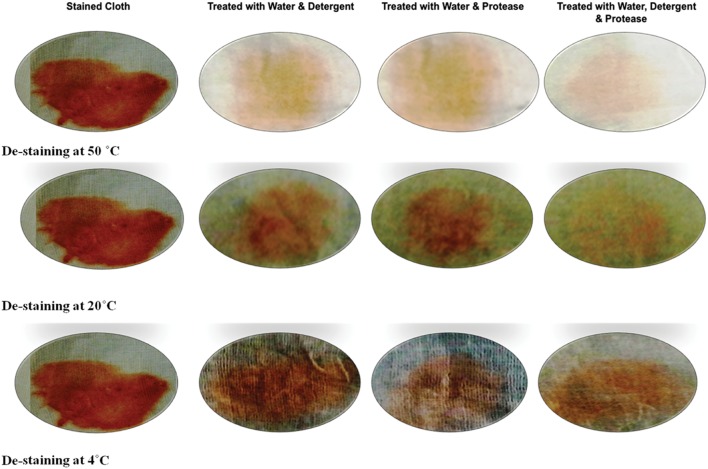
**Wash performance analysis of alkaline protease enzyme on different temperature**.

Washing performance of protease was estimated by treating the stained cloth at 4, 20, and 50°C for 15 min. The protease (109 U) exhibited variable de-staining performance at different temperature. The best de-staining was observed at temperature 50°C and there was considerable loss of stain even at 4°C as evident in **Figure 7.** The supplementation of protease with detergents showed better de-staining as compared to detergent alone. Moreover, protease alone has shown considerable de-staining ability. These observations support the hypothesis that the enzyme could be used as a detergent additive.

## Discussion

The demand for industrial enzymes have kept on increasing, thus it is inevitable to search novel enzymes that could withstand harsh industrial process conditions. In the present study, a protease producing *B. pumilus* MP 27 was isolated from Southern ocean. Though protease production by *Bacillus* sp. is well-known (Jayakumar et al., 2012), still the demand for isolation of efficient proteolytic strain with potent characteristics such as temperature tolerance and detergent stability has not been spurred. Moreover, 40% of cost in the production process is harnessed by the substrates; hence, it is obligatory to formulate media with cost-effective components (Joo et al., 2002). Proteases derived from the *Bacillus* have been exploited by various optimization studies (Maurer, 2004; Haddar et al., 2009; Jaouadi et al., 2010). However, to the best of our knowledge, this would be the first paper which deals with the optimization studies of protease production with wild *B. pumilus* strain isolated from Southern ocean. A few observations on thermotolerant protease have been reported in other bacterial species as shown in **Table 6.** But *B. pumilus* MP 27 was unique because it produced high titer of protease at 30°C.

**Table 6:** Comparision of protease activity from different bacterial species isolated at different temperatures.

Bacterial sp.	Source	Optimum incubation temperature (°C)	Optimum pH	Fermentation process	Protease yield (U/ml)	Reference
*Brevibacillus* sp. strain AS-S10-II	Soil	50	8	Smf	507	Rai and Mukherjee, 2011
*Bacillus subtilis* DR8806	Hot mineral spring	60	9.5	Smf	54.7	Asoodeh and Lagzian, 2012
*Bacillus mojavensis* A21	Marine water	60	8 and 10	Smf	250 in presence of detergent	Haddar et al., 2009
*Streptomyces* sp. DP2	Milk processing plants and slaughter houses	45	8	Smf	34.6	Bajaj and Sharma, 2011
*Bacillus pumilus* MP27	Southern Ocean	30	9	Smf	776.5	Present study


The key factors in the optimization of enzyme production are carbon and nitrogen sources that determine the overall production cost (Laxman et al., 2005). Among the various sources examined, wheat straw and peptone has showed the optimum activity. The agro-residues are complex substrates containing mixture of nutrients and their combined effect help to induce the protease activity. There are several reports where researchers showed enhanced production of various industrial enzymes by using different agro-residues. Since, soluble substrates like glucose, sucrose, etc. displayed very specific effect on cell growth and enzyme production. However, agro-residues like wheat and paddy straw, the single substrate itself contains multi-nutrients and helps to shoot up the enzyme production. The effect of various agro-residues on enzyme production may vary. Therefore, different agro-residues were screened to select the best one which can induce maximum protease enzyme production. Also the requirement of the particular substrate varies with the type of organisms and enzyme properties. There are few such reports Salihu et al., 2012).

The best nitrogen sources are organic in nature that support protease production since these serves as multinutrient factor that act as an inducer for protease production (Esakkiraj et al., 2011). This result was in agreement with that reported for marine *Bacillus* sp. MIG (Sanchez et al., 2003; Maurer, 2004) *Bacillus* sp. NPST-AK15 (Abdelnasser et al., 2015) and *Bacillus cluasii* (Lakshmi et al., 2014) where alkaline protease production was maximal using organic nitrogen source, as compared with inorganic nitrogen sources. Owing to the large availability and remarkable nutritional values, usage of such substrates may serve as a cost-effective source in the enzyme production as compared to expensive substrates like glucose (Jain et al., 2012), casein (Bouacem et al., 2015). Optimization of media components by this approach lead to the inexpensive constitution of media components, further, second step optimization, i.e., RSM evaluated the interaction among variables. It was reported that different metal ions have varied effect on the different types of proteases. The metal ions supporting the enzyme production for a particular enzyme might be inhibitory for other. It was reported by Haddar et al. (2009), Deng et al. (2010), Pillai et al. (2011) that calcium ions induces the enzyme production and also helps in thermostability of the protease. The surfactant was used to release the bound enzyme from the microbial cell wall. There might be some enzyme adhered to the wall which was released in the presence of the Tween-20 surfactant that might result into higher activity. When carbon and nitrogen sources were at their minima there is a significant drop in the protease activity. Therefore, it can be interpreted that interaction between carbon and nitrogen source had a severe impact on the protease production as evident from **Figure 3A.** This was also observed by Reddy et al. (2008). As evident, the carbon source played a lead role in the enhancement of the activity, since it was observed that the maximum concentration of carbon source produce better protease activity compared to all others. This result confirms earlier reports where the carbon source concentration was directly proportional to protease activity (Canan et al., 2006). The results were also validated by the *P*-value of the carbon source that is <0.0001. Further the interaction between the surfactant and carbon source is of relevance, where, the lower level in the carbon source was balanced by higher levels of surfactant for the establishment of worthy protease activity. This might be due to the adsorbed surfactant film around the cell which decreased or increased permeability or enhanced the availability of important ions, which has a favorable effect on protease activity (Mostafa et al., 2012). Thus, from the present study it can be concluded that protease production in *B. pumilus* can be improved by controlling the carbon source and surfactants and this interaction could only be well-understood by suitable RSM selection.

Designing of protease model *in silico* provided the relevant information about its active site and its interaction with various substrates by docking gave the best docking score. The protein was modeled with swiss model and the PDB ID of similar alkaline proteinase from the mesophilic bacterium *B. mesentericus* (1mee.1.A) was taken as template with 74.56% with query sequence and resolution of 2 Å. ProDom was employed for the detection of functional domain in modeled protein. ProDom is construction method based on iterative PSI-BLAST searches and multiple alignments are generated for each domain family. Serine-type endopeptidase activity was found in the translated sequence, the total length of conserve domain was 57 amino acids with total alignment score of 223 and Identities of 44/53 (83%), Positives of 48/53 (90%) with the target domain when submitted in the ProDom server. Such studies were also done in inulinases (Singh and Shukla, 2011; Singh et al., 2011, 2016), Xylanses (Karthik and Shukla, 2012).

For the application of protease as detergent additive, the enzyme must be alkali stable because the pH of the detergent lies in the range of 9–12 (Jain et al., 2012). The protease from *B. pumilus* is quite stable in alkaline range, though a maximum of 8. The other researchers also reported where thermotolerant alkaline protease was active under comparable range of pH (Banika and Prakash, 2004) and there are several reports on the optimization of enzymes, biological characterization of marine bacteria from Southern ocean, enzymatic applications, molecular modeling, bacterial platform technologies, systems biology approaches and gene editing tools which could be crucial toward improving the activity of enzyme and the industries now demand versatile proteases with activity at varied range of temperature (Shukla and Gupta, 2007; Shukla et al., 2007; Baweja et al., 2015; Gupta and Shukla, 2015, 2016a,b; Gupta et al., 2015; Karumuri et al., 2015; Singh and Shukla, 2015; Kumar et al., 2016). Although, the thermophilic proteases are efficient stain removers but the trend is now shifting toward cold washing since it minimizes the energy during heat up process. The protease currently studied has broad temperature stability, i.e., from 10 to 50°C. Also the wash performance analysis has depicted considerable de-staining at 4°C, though best at 50°C. Low temperature stability and washing performance study conclude it as novel protease for versatile detergent. The enzyme also showed remarkable compatibility with various surfactants and commercial detergents that completes its contribution as an effective laundry additive. Further, low level of supplementation, i.e., 109 U of protease in contrast with previously reported 845, 1690, 5070 U (Haddar et al., 2009; Jain et al., 2012), 500 U (Bouacem et al., 2015) along with broad temperature, pH range validates the superiority and novelty of protease enzyme produced by *B. pumilus* MP 27.

## Conclusion

**FIGURE 8:**
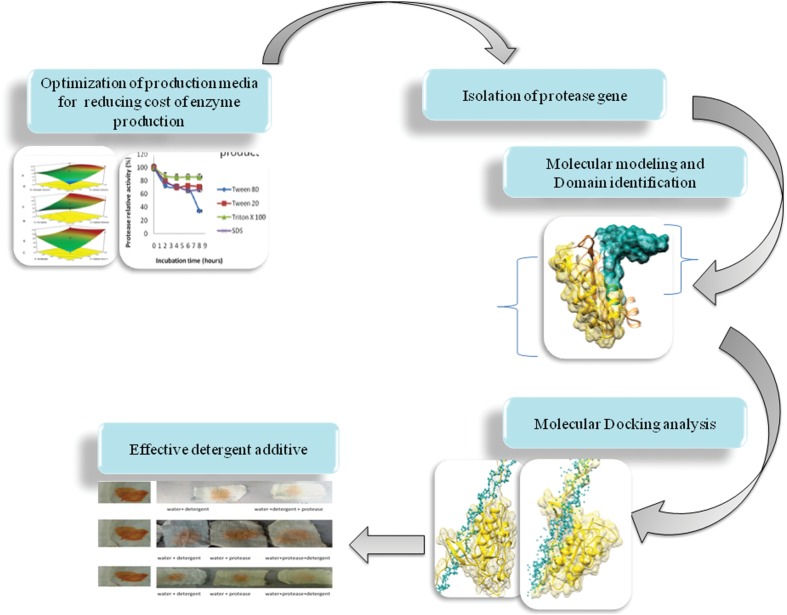
**Highlighted outcome of current work: a snapshot**.

The study reports isolation and selection of novel *B. pumilus* MP27 from Southern ocean. Selection of low cost substrates by two-step optimization, made the production process economical. Moreover, cost effective media components will further reduce industrial enzyme production cost. A snapshot of current work highlighting the outcome is depicted in **Figure 8.** Characterization of protease demonstrates its stability under broad temperature and pH range even in the presence of surfactants and commercial detergents. The wash performance analysis supported its candidature in detergent industry, even at low temperature at very low level of supplementation. Since enzyme was able to effectively remove food stain at low temperature, it can find application in cleaning sensitive clothes, which may not be washed at high temperature. Molecular modeling of the protease gene could enable us to learn about the catalytic behavior of the enzyme. Furthermore, molecular docking revealed the specificity of the presence of serine proteases, subtilase family and serine active site by docking with different substrate.

## Author Contributions

All authors listed, have made substantial, direct and intellectual contribution to the work, and approved it for publication.

## Conflict of Interest Statement

The authors declare that the research was conducted in the absence of any commercial or financial relationships that could be construed as a potential conflict of interest.

## Footnotes

1 http://prodom.prabi.fr/prodom/current/html/home.php

2 https://services.mbi.ucla.edu/SAVES/.
